# Ginsenoside Rg1 attenuates mitochondrial dysfunction in bleomycin-induced pulmonary fibrosis by inhibiting Nrf2/Sirt3 pathways

**DOI:** 10.1016/j.gendis.2026.102174

**Published:** 2026-04-02

**Authors:** Qi Qi, Shihao Wen, Linchao Niu, Ru Zhang, Meili Lu, Jingliang Zhang, Hongxin Wang

**Affiliations:** a Key Laboratory of Cardiovascular and Cerebrovascular Drug Research of Liaoning Province, Jinzhou Medical University, Jinzhou, Liaoning 121000, China; b Department of Anesthesiology, Jinzhou Central Hospital, Jinzhou Medical University Graduate Training Base, Jinzhou, Liaoning 121000, China; c Internal Medicine-Cardiovascular Department, The First Affiliated Hospital of Jinzhou Medical University, Jinzhou, Liaoning 121000, China

Idiopathic pulmonary fibrosis (IPF) constitutes a life-threatening form of interstitial lung disease.^1^ As the most common type of pulmonary fibrosis (PF), IPF is characterized by a poor prognosis, with a median survival of 2.5–3.5 years following diagnosis.^2^ The disease mechanism of IPF is not well understood. Main findings of current research are that persistent microinjury and abnormal repair of alveolar epithelial tissues caused oxidative stress, mitochondrial dysfunction, and epithelial–mesenchymal transition that promoted PF.^3^ Ginsenoside Rg1, a major active component of ginseng, has the characteristics of inhibiting inflammation, oxidative stress, and fibrosis, but the function and mechanism of ginsenoside Rg1 for PF are not clear.^4^ Nuclear factor E2-associated factor 2 (Nrf2), a key transcription factor regulating cellular antioxidant responses, was shown that the α subunit of Nrf2 combine directly with silent information regulator 3 (Sirt3), leading to the expression of Sirt3 mRNA.^5^ In this study, we demonstrated that ginsenoside Rg1 protected against PF by inhibiting collagen fiber deposition, oxidative stress, and mitochondrial dysfunction, and Nrf2 and Sirt3 may be the targets through which ginsenoside Rg1 ameliorates PF.

First, ginsenoside Rg1 administration elevated body weight and decreased the pulmonary index in PF mice (Fig. 1A; Fig. S1A). Hematoxylin-eosin staining results showed that the lung tissues in the bleomycin-treated (BLM) group were structurally disordered, with thickening of alveolar septa with breakage, alveolar atrophy, and massive proliferation of fibroblasts accompanied by extensive congestion and edema; compared with the BLM group, the alveolar structure of mice in the ginsenoside Rg1 group improved to different degrees, and the fibrosis symptoms were reduced (Fig. 1B). The Aschcroft scores of lung tissues in each ginsenoside Rg1 group were decreased significantly (Fig. S1B). During the course of the experiment, mice showed varying degrees of respiratory distress after modeling. Surfactant protein C (SP–C) lowers alveolar surface tension, which helps maintain the relative stability of alveolar volumes. Together with the immunofluorescence results, these findings suggest that ginsenoside Rg1 can increase SP-C protein content and stabilize alveolar size (Fig. S1C and S1D). The ELISA results showed that the levels of interleukin (IL)-1β, IL-6, and tumor necrosis factor-alpha (TNF-α) in the bronchoalveolar lavage fluid were significantly increased compared with those in control fluid; the IL-1β, IL-6, and TNF-α levels were lower after administration of ginsenoside Rg1 (Fig. S1E–S1G). The Masson and Sirius red staining showed that ginsenoside Rg1 could reduce the fiber deposition in lung tissues (Fig. 1C). The amount of hydroxyproline content responded to the amount of collagen deposition in lung tissue. Correspondingly, the level of hydroxyproline was down-regulated by ginsenoside Rg1 remarkably (Fig. 1D). α-smooth muscle actin (α-SMA) protein was increased in the BLM group, and the protein deposition was decreased by administration of ginsenoside Rg1 (Fig. S1H). Collectively, these study results suggest that ginsenoside Rg1 reduces collagen deposition and fibrosis in mice with PF.

**Figure 1:**
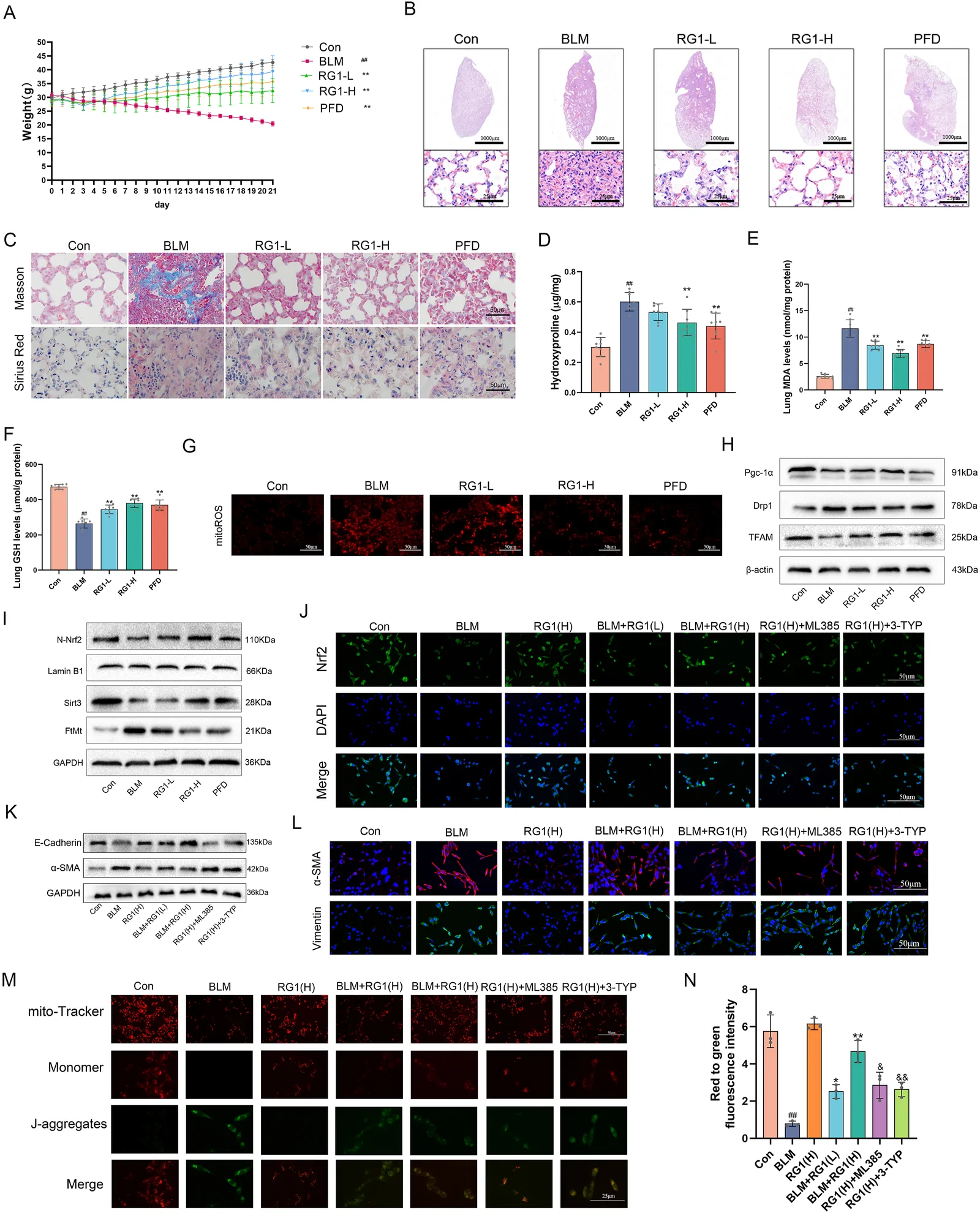
Ginsenoside Rg1 mediates mitochondrial dysfunction in BLM-induced pulmonary fibrosis by inhibiting Nrf2/Sirt3 pathways **(A)** The mouse body weight change (*n* = 10) **(B)** Lung tissue structural changes were examined by hematoxylin-eosin staining (*n* = 3) **(C)** Masson staining and Sirius red staining were performed for the detection of collagen deposition (*n* = 3) **(D)** The HYP content in lung tissue was detected by a kit (*n* = 8) **(E, F)** The levels of oxidative stress indicators MDA and GSH in lung tissue were measured by a kit (*n* = 8) **(G)** Fluorescent probes were used to detect the production of mitoROS in lung tissue (*n* = 3) **(H)** The protein levels of Drp1, Pgc-1α, and TFAM were detected by Western blotting (*n* = 3) **(I)** The protein levels of N-Nrf2, Sirt3, and FtMt were detected by Western blotting (*n* = 3) **(J)** Immunofluorescence staining was used to detect the expression of Nrf2 in cells (*n* = 3) **(K)** The protein levels of α-SMA and E-Cadherin were detected by Western blotting (*n* = 3) **(L)** Immunofluorescence staining was used to detect the expression of α-SMA and Vimentin proteins in cells (*n* = 3) **(M,** N**)** Mitochondria and mitochondrial membrane potential were examined by fluorescent probes (*n* = 3). Results were presented as mean ± standard deviation. ^##^*p* < 0.01 compared with the control group; ∗*p* < 0.05 and ∗∗*p* < 0.01 compared with the BLM group; ^&^*p* < 0.05 and ^&&^*p* < 0.01 compared with the BLM + RG1(H) group. RG1, ginsenoside Rg1; BLM, bleomycin; HYP, hydroxyproline; MDA, malondialdehyde; GSH, glutathione; mitoROS, mitochondrial reactive oxygen species; Pgc-1α, peroxisome proliferator-activated receptor-gamma coactivator-1α; Drp1, dynamin-related protein 1; TFAM, mitochondrial transcription factor A; Nrf2, nuclear factor erythroid 2-related factor 2; Sirt3, silent information regulator 3; FtMt, ferritin mitochondrial; E-Cadherin, epithelial cadherin; α-SMA, α-smooth muscle actin.

Excessive reactive oxygen species (ROS) buildup brought on by intracellular iron overload leads to oxidative stress. When intracellular Fe^2+^ levels increase, large amounts of ROS are produced via the Fenton reaction, indicating the occurrence of oxidative stress. Prussian blue staining showed that after BLM administration, a large number of brown iron particles were deposited in the lung tissues of mice, distributed along the alveolar septa. When ginsenoside Rg1 was administered, the amount of brownish iron particles was lower than in the BLM group (Fig. S2A). We then measured Fe^2+^ and ROS contents in lung tissues, demonstrating that ginsenoside Rg1 decreased Fe^2+^ and ROS levels in PF mice (Fig. S2B–S2D). Mice in the BLM group had significantly higher malondialdehyde (MDA) levels and diminished glutathione (GSH), catalase (CAT), and superoxide dismutase (SOD) levels, while those in the ginsenoside Rg1 group presented with lower MDA levels and increased levels of GSH, CAT, and SOD (Fig. 1E and F; Fig. S2E and S2F). These suggest that ginsenoside Rg1 can effectively reduce the deposition of Fe^2+^ in lung tissue stimulated by BLM, which in turn reduces the ROS and inhibits oxidative stress. Mitochondrial ROS (mitoROS) reflects the extent of mitochondrial damage to a certain extent. The fluorescence of mitoROS in the BLM group was increased, and mitoROS decreased significantly after ginsenoside Rg1 intervention (Fig. 1G). Dynamin-related protein 1 (Drp1) regulates mitochondrial division, and peroxisome proliferator-activated receptor-gamma coactivator-1 alpha (Pgc-1α) and mitochondrial transcription factor A (TFAM) proteins promote mitochondrial synthesis. The balance of these three proteins collectively maintains mitochondrial dynamic stability. The result of Western blotting showed that after BLM administration, Drp1 protein expression was significantly increased, and mitochondrial abnormal fission was increased, which is not conducive to the function of mitochondria. After ginsenoside Rg1 intervention, the expression of these proteins was restored, and the stability of mitochondrial dynamics was maintained (Fig. 1H).

We analyzed the possible mechanism through which ginsenoside Rg1 inhibited PF. Ginsenoside Rg1-related targets were obtained from the CAT database and the Swiss Target Prediction database, and then IPF-related targets were obtained from the GeneCards database and the CTD database. Next, 133 common targets shared by ginsenoside Rg1 and IPF were obtained (Fig. S3A). Kyoto Encyclopedia of Genes and Genomes (KEGG) and Gene Ontology (GO) pathways were analyzed for the 133 targets (Fig. S3B and S3C). Among the 133 proteins, NFE2L2 (nuclear factor erythroid 2-related factor 2, Nrf2) in particular aroused our interest since the molecule regulates mitochondrial dysfunction and oxidative stress, which are closely related to the occurrence and development of IPF (Fig. S3D). After reviewing the literature and previous reports, we identified the downstream proteins of Nrf2, Sirt3, and mitochondrial ferritin (FtMt). Therefore, we investigated the effects of ginsenoside Rg1 on Nrf2 and its downstream proteins Sirt3 and FtMt. Molecular docking studies revealed that ginsenoside Rg1 interacted with Nrf2, Sirt3, and FtMt with favorable docking scores (Fig. S3E–S3H). The Western blotting results showed that the Nrf2 and Sirt3 proteins in the BLM group were significantly decreased, and the expression of FtMt protein was significantly increased; administration of ginsenoside Rg1 reversed these changes (Fig. 1I). Consistent results were observed in immunohistochemical analyses (Fig. S3I–S3K). *In vitro*, the fluorescence-positive intensity expression of Nrf2 protein was reduced in the BLM group; Nrf2 fluorescence-positive intensity expression was increased in the ginsenoside Rg1 administered group, and the administration of ML385 (Nrf2 inhibitor) and 3-TYP (Sirt3 inhibitor) partially reversed the above changes (Fig. 1J). In general, ginsenoside Rg1 plays a protective role in PF through the Nrf2/Sirt3 pathways.

One of the driving forces behind PF is epithelial–mesenchymal transition. At the cellular level, decreased E-Cadherin expression and increased α-SMA expression indicated that A549 cells underwent epithelial–mesenchymal transition following BLM treatment, which was reversed by ginsenoside Rg1, possibly via the Nrf2/Sirt3 signaling pathway. Cellular immunofluorescence analysis showed that α-SMA and Vimentin fluorescence intensity were increased in the BLM group; in contrast, ginsenoside Rg1 administration reduced the fluorescence intensity of these two proteins, and co-administration of ML385 or 3-TYP partially reversed these effects (Fig. 1K and L). In the BLM group, mitochondrial number was significantly decreased, and mitochondrial membrane potential was down-regulated; whereas ginsenoside Rg1 treatment increased mitochondrial number and restored mitochondrial membrane potential, and these changes were partially reversed by ML385 or 3-TYP administration (Fig. 1M and N).

In this study, ginsenoside Rg1 ameliorates PF by inhibiting collagen deposition, oxidative stress, and mitochondrial dysfunction. These findings may provide new insights into the treatment of PF.

## CRediT authorship contribution statement

**Qi Qi:** Writing – review & editing, Writing – original draft, Visualization, Data curation, Conceptualization. **Shihao Wen:** Writing – original draft. **Linchao Niu:** Writing – original draft. **Ru Zhang:** Visualization. **Meili Lu:** Writing – review & editing, Writing – original draft. **Jingliang Zhang:** Writing – review & editing, Writing – original draft. **Hongxin Wang:** Writing – review & editing, Writing – original draft.

## Data availability

The data will be made available upon request.

## Ethics declaration

The experiment was conducted in accordance with the ethical review standards of Jinzhou Medical University.

## Funding

This work was supported by the 10.13039/501100001809National Natural Science Foundation of China (No. 82474170).

## Conflict of interests

The authors declared no conflict of interests that could have appeared to influence the work reported in this paper.

## References

[1] Moss B.J., Ryter S.W., Rosas I.O. (2022). Pathogenic mechanisms underlying idiopathic pulmonary fibrosis. Annu Rev Pathol.

[2] Shao L., Zhang Y., Shi W. (2021). Mesenchymal stromal cells can repair radiation-induced pulmonary fibrosis via a DKK-1-mediated Wnt/β-catenin pathway. Cell Tissue Res.

[3] Podolanczuk A.J., Thomson C.C., Remy-Jardin M. (2023). Idiopathic pulmonary fibrosis: state of the art for 2023. Eur Respir J.

[4] Yang S.J., Wang J.J., Cheng P., Chen L.X., Hu J.M., Zhu G.Q. (2023). Ginsenoside Rg1 in neurological diseases: from bench to bedside. Acta Pharmacol Sin.

[5] Satterstrom F.K., Swindell W.R., Laurent G., Vyas S., Bulyk M.L., Haigis M.C. (2015). Nuclear respiratory factor 2 induces SIRT3 expression. Aging Cell.

