# Supplementary material for: Ginsenoside Rg1 attenuates mitochondrial dysfunction in bleomycin-induced pulmonary fibrosis by inhibiting Nrf2/Sirt3 pathways
Source: Genes Dis. 2026 Apr 2;14(1):102174. doi: 10.1016/j.gendis.2026.102174 (PMC13545364; doi:10.1016/j.gendis.2026.102174)
Supplement: Supplementary file 1 [file mmc1.docx]

Ginsenoside Rg1 attenuates mitochondrial dysfunction in bleomycin-induced pulmonary fibrosis by inhibiting Nrf2/Sirt3 pathways

**Abstract**

**Background:** Mitochondrial dysfunction promotes the development of pulmonary fibrosis (PF), whereas ginsenoside Rg1 ameliorates respiratory diseases. Whether ginsenoside Rg1 can alleviate bleomycin-induced PF remains unknown. **Methods:** In this study, the potential targets of ginsenoside Rg1 in PF were investigated using network pharmacology and molecular docking methods. PF mouse model was established by a single intratracheal instillation of bleomycin (BLM). Human lung adenocarcinoma (A549) cells were injured with BLM and then treated with ginsenoside Rg1. To evaluate the therapeutic effects of ginsenoside Rg1, this study assessed indicators related to inflammatory responses, mitochondrial dysfunction and oxidative stress. **Results:** The results indicated that ginsenoside Rg1 improved the survival and body weight of PF mice while reducing the levels of the inflammatory factors IL-1β, TNF-α, and IL-6. Ginsenoside Rg1 alleviated alveolar wall thickening and collagen fiber deposition in PF mice, reduced the levels of hydroxyproline (HYP) and malondialdehyde (MDA), increased glutathione (GSH) expression, and attenuated oxidative stress. In addition, ginsenoside Rg1 attenuated bleomycin-induced mitochondrial dysfunction in PF by upregulating Pgc-1α and TFAM protein expression and downregulating Drp1 production. Importantly, ginsenoside Rg1 significantly reversed the bleomycin-induced downregulation of Nrf2 and Sirt3 protein expression. In vitro studies showed that Nrf2 inhibitors and Sirt3 inhibitors abrogated the ginsenoside Rg1-induced upregulation of Nrf2 and Sirt3 proteins. **Conclusion:** In this study, ginsenoside Rg1 protects against PF by inhibiting collagen fiber deposition, oxidative stress, and mitochondrial dysfunction, and Nrf2 and Sirt3 may be the key targets through which ginsenoside Rg1 ameliorates PF.

**Keywords:** Pulmonary fibrosis; Ginsenoside Rg1; Mitochondrial dysfunction; Oxidative stress

**Supplementary Figure1. Ginsenoside Rg1 improves survival in mice with pulmonary fibrosis.** (A) Pulmonary index (n=8). (B) The Ashcroft scores of lung tissue were evaluated according to HE staining results. (C-D) Lung tissue was subjected to SP-C immunofluorescence staining, with quantitative analysis of the fluorescence intensity (n=3). (E-G) The IL-1β, IL-6 and TNF-α levels in BALF were determined and statistically analyzed (n=8). (H) The expression of α-SMA protein in lung tissue was examined by immunohistochemistry (n=3). Results were presented as mean ± standard deviation. ^##^*P*＜0.01 compared with the control group; ^*^*P*＜0.05, ^**^*P*＜0.01 compared with the BLM group. *P*＜0.01, *P*＜0.05 indicates a significant difference between the indicated groups. SP-C: surfactant protein C; IL-1β: interleukin-1β; IL-6: interleukin-6; TNF-α: tumor necrosis factor-α; α-SMA: α-smooth muscle actin.

**Supplementary Figure 2 Ginsenoside Rg1 reduces oxidative stress in mice with pulmonary fibrosis.** (A) Prussian blue staining was used to detect iron deposition in lung tissue (n=3). (B) The level of Fe^2+^ content in lung tissue (n=8). (C-D) Detection of reactive oxygen species (ROS) generation using fluorescent probes and quantitative analysis of fluorescence intensity (n=3). (E-F) CAT and SOD levels in lung tissues were measured with corresponding detection kits (n=8). Data are expressed as the mean ± SD. ^##^*P*＜0.01 compared with the control group; ^*^*P*＜0.05, ^**^*P*＜0.01 compared with the BLM group. *P*＜0.01, *P*＜0.05 indicates a significant difference between the indicated groups. ROS: reactive oxygen species; CAT: catalase; SOD: superoxide dismutase.

.

**Supplementary Figure 3 Ginsenoside Rg1 activates the Nrf2/Sirt3 signaling pathway in mice with pulmonary fibrosis.** (A) Rg1- IPF targets Wayne figure. (B) KEGG enrichment analysis bubble diagram. (C) GO enrichment analysis histogram. (D) PPI network diagram. (E) NFE2L2 (PDB: 6qmc) - ginsenoside Rg1 (MOL011401). (F) Sirt3 (PDB: 8v2n) - ginsenoside Rg1 (MOL011401). (G) FtMt (PDB: 7o63) - ginsenoside Rg1 (MOL011401). (H) NFE2LE, Sirt3 and FtMt with ginsenoside Rg1 docking score (kcal/mol). (I-K) The expression of Nrf2 and Sirt3 was examined by immunohistochemical staining (n=3). Data are expressed as the mean ± SD. ^##^*P*＜0.01 compared with the control group; ^*^*P*＜0.05, ^**^*P*＜0.01 compared with the BLM group. *P*＜0.01, *P*＜0.05 indicates a significant difference between the indicated groups. Nrf2: nuclear factor erythroid 2-related factor 2; Sirt3: silent information regulator 3.

**Materials and methods**

**Reagents and antibodies**

Bleomycin (Selleck); Pirfenidone (Beijing Continent Pharmaceuticals Co., Ltd.); Nuclear factor E2 associated factor 2 (Nrf2, Cat#16396-1-AP), Sirtuin 3 (Sirt3, Cat#10099-1-AP), Peroxisome proliferator activated receptor gamma coactivator 1 alpha (Pgc-1α, Cat#66369-1-Ig), Dynamin related protein 1 (Drp1, Cat#12957-1-AP) and Mitochondrial transcription factor A (TFAM, Cat#22586-1-AP)(Proteintech, wuhan). Mitochondrial ferritin (FtMt, Cat#A12867), E-Cadherin (Cat#A20798), α-SMA (Cat#A2235), β-actin (Cat#AC038), GAPDH (Cat#A19056), Lamin B1 (Cat#A11495), Vimentin (Cat#A19607) and HRP-conjugated goat anti-rabbit secondary antibodies (Cat#AS014)(ABclonal, wuhan). Hydroxyproline (HYP), Tissue iron (Fe^2+^), Malondialdehyde (MDA), Superoxide dismutase (SOD), Reduced glutathione (GSH) and Catalase (CAT) kits (Jiancheng Bioengineering Institute, Nanjing, China). Tumor necrosis factor-ɑ (TNF-ɑ), interleukin-1β (IL-1β) and interleukin-6 (IL-6) ELISA kits (EK-Bioscience, enzyme research, Shanghai, China).

**Network pharmacology**

The ginsenoside Rg1-related targets were obtained from the CTD database (https://ctdbase.org/) and SwissTargetPrediction database (https://www.swisstargetprediction.ch/). The IPF-related targets were collected from the GeneCards database (www.genecards.org/) and CTD database using "Idiopathic pulmonary fibrosis" as the keyword. Afterward, the intersection between Rg1-related and IPF-related targets was identified by the online Venn diagram tool (https://bioinfogp.cnb.csic.es/tools/venny/index.html/). Next, the targets were introduced into the STRING online platform (https://string-db.org/) to construct a protein-protein interaction network. In order to ensure good accuracy of the targets, we chose a confidence level of 0.4 as the analysis criterion. Then, the analysis results were imported into Cytoscape 3.9.1. The node size was set according to the degree value to obtain a network graph of interactions between protein targets. Finally, the key targets were imported into the Metascape database (https://www.metascape.org) for GO function and KEGG pathway enrichment analyses.

**Molecular docking of ginsenoside Rg1 and key targets**

Protein molecule structures were downloaded from the PDB database (https://www.rcsb.org/), and PyMOL was used to process the protein molecules, including removing water molecules and adding hydrogen ions. The 3D structure of ginsenoside Rg1 was downloaded from the PubChem database (https://pubchem.ncbi.nlm.nih.gov/). AutoDock was used to perform molecular docking between target proteins and ginsenoside Rg1. The docking score represents the binding free energy; the higher the binding free energy, the more stable the binding between ginsenoside Rg1 and the receptor. The docking results were visualized using PyMOL 2.5.

**Animals and treatments**

Seventy-five C57BL/6 mice (20-25 g), 5-6 weeks of age, were provided by the Animal Experimental Center of Jinzhou Medical University. The experiment was conducted in accordance with the ethical review standards of Jinzhou Medical University. A pulmonary fibrosis model was established in mice by referring to the method described in the literature. Mice were anesthetized with sodium pentobarbital (35 mg/kg) via intraperitoneal injection, then fixed in the supine position and administered bleomycin (3.5 mg/kg) via intratracheal instillation. Mice in the control group were instilled with normal saline. On the day after modeling, the mice were randomly divided into 5 groups: control (Con), model (BLM), low-dose ginsenoside Rg1 (RG1-L, 10 mg/kg), high-dose ginsenoside Rg1 (RG1-H, 20 mg/kg), and pirfenidone (PFD, 100 mg/kg), with 15 mice per group. Mice in the ginsenoside Rg1 groups and pirfenidone group were given 0.2 mL of the corresponding drug by gavage once daily for 21 consecutive days. The control (Con) and model (BLM) groups were administered an equal volume of 0.5% sodium carboxymethylcellulose (CMC-Na) via gavage. On day 22, blood samples and lung tissues were collected for subsequent experiments.

**Cell and treatment**

Human lung adenocarcinoma cells (A549 cells, Pricella, Wuhan) were cultured in DMEM supplemented with fetal bovine serum, penicillin (100 U/mL), and streptomycin (100 μg/mL) in an incubator at 37 °C with 5% CO₂. When the cells reached approximately 80% confluency, they were trypsinized and passaged, and cells in the logarithmic growth phase were used for the experiments. Groups were as follow: (1): control group (Con); (2): model group (BLM): bleomycin (40 μg/mL); (3): Rg1(H) group (RG1(H)): ginsenoside Rg1 (20 μmol/L); (4): model + Rg1(L) group (BLM+RG1(L): ginsenoside Rg1 (10 μmol/L); (5): model + Rg1(H) group (BLM+RG1(H); (6): model + Rg1(H) + ML385 group (RG1(H)+ML385): ML384(10 μmol/L); (7): model + Rg1(H) + 3-TYP group (RG1(H)+3-TYP): 3-TYP (1 μmol/L). Part of the cells and the supernatant were collected for subsequent experiments.

**Determination of the rate of change in body weight and pulmonary index**

The body weight of mice was recorded daily. After the mice were euthanized, the chest was opened and the lungs were removed. Filter paper was used to absorb surface moisture, and the lungs were then weighed (mg). The pulmonary index (mg/g) was calculated as follows:

Pulmonary index (mg/g) = lung weight (mg) / body weight (g).

**Determination of cytokines levels in BALF**

After the neck tissues were separated and exposed the trachea, used a 20 G cannula inserted into the main bronchus and irrigated with saline for 3 times to collect the BALF. The BALF was centrifuged at 5000 rpm/5 min, and the supernatant was collected. According to the kit instructions tested the TNF-α, IL-6, and IL-1β levels.

**Histopathology**

Mouse lung tissues were fixed in 4% paraformaldehyde solution, followed by dehydration in graded alcohols (from low to high concentration), clearing in xylene twice, embedding in paraffin blocks, and sectioning into 5 μm-thick slices. Paraffin sections were subjected to HE, Masson, Sirius Red, and Prussian blue staining. After dehydration and mounting, the sections were observed under a microscope to assess histopathological changes in the lung tissues.

**Immunohistochemistry**

Paraffin sections were baked in an oven for 2 h, then deparaffinized in xylene for 30 min. Subsequently, the sections were hydrated in a graded ethanol series (100%, 95%, 90%, 80%, 70%), rinsed with PBS, and subjected to antigen retrieval according to the kit instructions. Endogenous peroxidase blocker was added, and the sections were incubated at room temperature for 10 min, followed by washing with PBS for 3 min (3 times). Primary antibody was added, and the sections were incubated overnight at 4 °C, then washed with PBS. Enzyme-labeled goat anti-rabbit IgG polymer was added and incubated for 20 min, followed by DAB chromogenic reaction and hematoxylin counterstaining for nuclei. The sections were then dehydrated in graded ethanol, cleared in xylene, and mounted with resin. Positive expression was identified as brownish-yellow particles under a microscope.

**Immunofluorescence staining**

Paraffin sections were baked at 60 °C for 2 h, followed by deparaffinization and hydration. Antigen retrieval was then performed, and the sections were permeabilized with 0.5% Triton X-100 for 1 h, rinsed with PBS for 3 min (3 times). Non-specific binding was blocked with 5% bovine serum albumin (BSA) for 1 h at room temperature. Diluted primary antibody was added dropwise, and the sections were incubated overnight at 4 °C. The next day, unbound primary antibody was washed away with PBS, and the sections were incubated with fluorescent secondary antibody for 3 h in the dark. Nuclei were stained with DAPI, and the sections were mounted with anti-fluorescence quenching medium. Images were captured using a fluorescence microscope.

**Determination of oxidative stress and HYP and Fe^2+^ contents**

Appropriate amount of lung tissue was ground to obtain the supernatant for experiments. The levels of GSH, MDA, CAT, SOD, HYP, and Fe^2+^ in lung tissues were determined using GSH, MDA, CAT, SOD, HYP, and Fe^2+^ assay kits.

**ROS, mitochondrial ROS and mitochondria fluorescent probe assay**

Paraffin sections were baked at 60 °C for 2 h, then dewaxed to water. DHE staining solution was added, and the sections were incubated at 37 °C for 30 min. After washing with PBS for 5 min (3 times), nuclei were stained with DAPI. Under a fluorescence microscope, red fluorescence indicated a positive result. The results were analyzed using Image J. For mitochondrial ROS staining, 5 μM working solution was added to cover the sectioned tissues or cells, followed by incubation at 37 °C for 10 min. After washing with PBS for 5 min (3 times) and nuclear staining, the samples were observed under a fluorescence microscope, with red fluorescence considered a positive result.

**Mitochondrial membrane potential evaluation**

The mitochondrial membrane potential of A549 was assessed using JC-1 staining (Beyotime Biotechnology, Shanghai, China). 10 μg/mL JC-1 staining solution was added to the treated cells and incubated in the dark for 20 min. Then, the cells were washed with PBS and the images were captured by fluorescence microscopy.

**Western blot**

Proteins were extracted from A549 cells and lung tissues using RIPA lysis buffer containing protease inhibitors. Nuclear and mitochondrial proteins were extracted from tissues or cells according to the instructions of the Nuclear and Mitochondrial Protein Extraction Kit. Protein concentrations were determined using the BCA method. Approximately 20 μg of protein samples were loaded per well and separated by 10% or 12% SDS-PAGE. The initial electrophoresis voltage was 80 V, and the voltage was adjusted by monitoring the migration of the protein marker; electrophoresis was terminated at an appropriate time. After electrophoresis, the proteins were transferred to PVDF membranes, which were then blocked with 5% skim milk for 1.5 h. The PVDF membranes were incubated with primary antibodies at 4 °C overnight. The primary antibodies used were as follows: Nrf2, Sirt3, FtMt, Pgc-1α, Drp1, TFAM, E-cadherin, Vimentin, α-SMA, β-actin, GAPDH, and Lamin B1. On the following day, the PVDF membranes were washed three times with TBST, then incubated with secondary antibodies on a shaker for 1.5 h. After washing three times with TBST, ECL developer was prepared by mixing solutions A and B at a 1:1 ratio, and protein bands were detected using a gel imaging system.

**Statistical analysis**

Sample sizes for animal and cell experiments were determined by statistical power analysis using G*Power 3.1 software. GraphPad Prism 8.0 software was used to perform One-way ANOVA analysis and to graph. Data were presented as the mean ± standard deviation (SD). *P*＜0.05 was considered statistically significant.
